# Consistency and stability of individualized cortical functional networks parcellation at 3.0 T and 5.0 T MRI

**DOI:** 10.3389/fnins.2024.1425032

**Published:** 2024-08-19

**Authors:** Minhua Yu, Bo Rao, Yayun Cao, Lei Gao, Huan Li, Xiaopeng Song, Haibo Xu

**Affiliations:** ^1^Department of Radiology, Zhongnan Hospital of Wuhan University, Wuhan, China; ^2^Central Research Institute, United Imaging Healthcare, Shanghai, China

**Keywords:** 5.0 T, resting-state fMRI, individualized parcellation, sub-networks, consistency, stability

## Abstract

**Background:**

Individualized cortical functional networks parcellation has been reported as highly reproducible at 3.0 T. However, in view of the complexity of cortical networks and the greatly increased sensitivity provided by ultra-high field 5.0 T MRI, the parcellation consistency between different magnetic fields is unclear.

**Purpose:**

To explore the consistency and stability of individualized cortical functional networks parcellation at 3.0 T and 5.0 T MRI based on spatial and functional connectivity analysis.

**Materials and methods:**

Thirty healthy young participants were enrolled. Each subject underwent resting-state fMRI at both 3.0 T and 5.0 T in a random order in less than 48 h. The individualized cortical functional networks was parcellated for each subject using a previously proposed iteration algorithm. Dice coefficient was used to evaluate the spatial consistency of parcellated networks between 3.0 T and 5.0 T. Functional connectivity (FC) consistency was evaluated using the Euclidian distance and Graph-theory metrics.

**Results:**

A functional cortical atlas consisting of 18 networks was individually parcellated at 3.0 T and 5.0 T. The spatial consistency of these networks at 3.0 T and 5.0 T for the same subject was significantly higher than that of inter-individuals. The FC between the 18 networks acquired at 3.0 T and 5.0 T were highly consistent for the same subject. Positive cross-subject correlations in Graph-theory metrics were found between 3.0 T and 5.0 T.

**Conclusion:**

Individualized cortical functional networks at 3.0 T and 5.0 T showed consistent and stable parcellation results both spatially and functionally. The 5.0 T MR provides finer functional sub-network characteristics than that of 3.0 T.

## Introduction

The human cerebral cortex consist of hundreds of cortical regions with unique structural morphology, functionality and connectivity (Kaas, 1987; Felleman and Van Essen, 1991; Zilles and Amunts, 2010; Eickhoff et al., 2018). The highly folded structure of cerebral cortex and multiple areas are thought to be organized into numerous spatially distributed large-scale networks, that interact as part of distributed functional networks and broadly serve for different aspects of human cognition and behavior (Fischl and Dale, 2000; Bressler and Menon, 2010). However, individualized differences in brain organization are distributed heterogeneously across the cortex and white matter fiber tracts (Wang et al., 2015; Han et al., 2020), contributing significantly to the complexity of the cortical networks and individual variability. Most previous studies of functional networks have focused on the population-level or group-level, failing to fully account for individual differences. Applying a group-template to individual subject may bring misalignment, dilute brain-behavior associations with neuropsychiatric diseases, obscure individual differences in the calculation process and restrict the development of personalized medical treatment in clinical practice (Wang et al., 2015). Obtaining functional atlases at the individual level is an important step to explore individual brain function and thus can further provide basis for precision personalized medicine.

Wang et al. developed a novel cortical parcellation approach to accurately map functional organization at the individual level using resting-state blood oxygenation level dependent functional MRI (BOLD-fMRI) (Wang et al., 2015). A population-based functional atlas and a map of inter-individual variability were employed to guide the iterative search for functional networks in individual subjects. Functional networks mapped by this approach were highly reproducible within subjects and effectively captured the variability across subjects (Wang et al., 2015). Functional connectivity (FC) is defined as the temporal dependency of neurophysiological signals, such as BOLD-fMRI signals, between spatially remote brain areas (Friston et al., 1993). Individual-specific functional connectivity analysis may contribute to reflecting individual characteristics of diseases, revealing the pathogenesis of diseases, and providing the possibility for early diagnosis and treatment monitoring.

It is generally known that MRI with high magnetic field can provide more detailed anatomical information relying on higher spatial resolution and signal-to-noise ratio compared to lower fields MRI (Triantafyllou et al., 2005; Schafer et al., 2008; de Hollander et al., 2017). The BOLD signal arises from the inhomogeneity of the local field, which is caused by the difference in magnetic susceptibility between the deoxygenated hemoglobin-rich blood in the capillaries and venous vessels and the surrounding tissues. For resting-state fMRI, the ultra-high field strength MRI greatly increases sensitivity to the BOLD contrast (Triantafyllou et al., 2005). Recently, a 5.0 T MRI scanner was developed. The high field strengths of 5.0 T may provide more accurate topological and functional maps at individual level than that of 3.0 T (Prudent et al., 2010).

The individualized cortical functional networks parcellation method proposed by Wang et al. has been reported as highly reproducible at 3.0 T (Wang et al., 2015). However, this method has never been tested on high field MRI above 3.0 T, such as 5.0 T MRI. It remains unclear whether the spatial distribution of these functional networks for the same subject would remain similar at different magnetic fields. Given the complexity of cortical networks and the significantly increased sensitivity to BOLD effects at ultra-high fields, this study aimed to explore the consistency and stability of individualized cortical functional networks parcellation across 3.0 T and 5.0 T MRI based on spatial measurement, between-network functional connectivity, and graph-theory analysis.

## Materials and methods

### Participants

This prospective study was approved by the Medical Research Ethics Committee and Institutional Review Board of Zhongnan Hospital and written informed consent was obtained from all subjects. Thirty healthy young individuals (mean age ± SD, 27 years ±5; male: 16, female: 14) were enrolled from September 2021 to March 2022. All participants were right-handed and without any comorbidities.

### MR acquisitions

Each participant underwent both 3.0 T and 5.0 T MRI scan in a random order within a 48-h window. The imaging protocols included resting-state blood oxygenation level dependent (rs-BOLD) MRI and structural three-dimensional (3D) T1-weighted images. The 3.0 T examinations were conducted with a commercial MR scanner (Discovery MR 750w, GE Healthcare, Waukesha, WI, United States) equipped with a 32-channel head coil. The 5.0 T examinations were performed with a newly MR scanner (uMR Jupiter, United Imaging Healthcare, Shanghai, China), also with a 32-channel head coil. To ensure homogeneous and reliable results, the main parameters were standardized between 3.0 T and 5.0 T devices. Resting-state data were acquired using a gradient echo-planar imaging (EPI) sequence with the following parameters: TR/TE = 2000/25 ms, timepoints = 240, FOV = 240 mm × 240 mm, flip angle (FA) = 90^°^, Acquisition Matrix = 60 × 60, slice thickness = 4.0 mm, number of slices = 39, and voxel size = 4 × 4 × 4 mm3. Structural 3D T1-weighted images for the 3.0 T scans were acquired with a brain volume imaging (BRAVO) sequence: TR/TE = 8.0/3.0 ms, thickness = 1.0 mm, FA = 12^°^, Acquisition Matrix = 240 × 240, FOV = 240 mm × 240 mm, number of slices = 148 and voxel size = 1 × 1 × 1 mm^3^. For the 5.0 T scans, structural 3D T1-weighted images were acquired with a three-dimension fast spoiled gradient echo sequence (T1 GRE-FSP 3D) with identical parameter to the 3.0 T scans. During resting functional MRI scanning, participants were instructed to relax, lie down comfortably with eyes closed, remain awake, and minimize cognitive activity. Head motion was minimized using foam pads, and earplugs were provided to attenuate scanner noise, ensuring smooth progress of the MRI scan and image quality.

### SNR, tSNR and CNR of raw resting-state fMRI data evaluation

To compare the Signal-to-Noise Ratio (SNR) of 3.0 T and 5.0 T, the signal component (S) was determined as the mean intensity within the selected parenchyma region of interests (ROIs). The ROIs were symmetrically drawn in frontal and parietal lobes at the apex level of the lateral ventricle from axial conventional resting-state fMRI data (Supplementary Figure S1). The noise (N) area was defined as regions in the four corners of the image, and its standard deviation was determined by calculating the mean of the standard deviation of four corners (Han et al., 2021). The mean SNR was calculated by S/N. The temporal SNR (tSNR) was computed on a voxel-wise basis and defined as the temporal mean signal divided by temporal standard deviation in subjects. Considering that neuronal activity signals may lead to bias in the measurement of tSNR on BOLD images, we also scanned a water phantom at 3.0 T and 5.0 T MR and calculated the tSNR, respectively. For Contrast-to-Noise Ratio (CNR) of 3.0 T and 5.0 T, we collected the mean intensity of gray (S_gray_) and white matter (S_white_). The ROI of gray matter was drawn on the frontal lobe at the apex level of the lateral ventricle from axial raw resting-state fMRI, and ROI of white matter was drawn in callosum area of the same level as gray matter ROI (Supplementary Figure S1). The CNR was calculated by (S_gray_-S_white_)/S_white_.

### fMRI preprocessing

Resting-state fMRI data, preprocessed using the Independent Component Analysis Fix (ICA-FIX) method from Human Connectome Project (HCP) subjects and presented as temporal series of grayordinates, were processed using the HCP pipeline (Glasser et al., 2016). The preprocessing steps involved the use of FSL (FMRIB Software Library), FreeSurfer, and command line functions from Connectome Workbench (Marcus et al., 2011; Barch et al., 2013; Smith et al., 2013; Glasser et al., 2016). Resting-state fMRI data for each subject underwent resampling to a standardized cortical surface mesh representation (fs_LR 32 k mesh). Despite the application of ICA-FIX, previous studies noted that global physiological noise and motion-related artifacts were not entirely eliminated (Marcus et al., 2011; Glasser et al., 2016). To enhance the resting-state fMRI analysis, we implemented additional processing steps:(1) Normalization of resting-state fMRI time series at each vertex to zero mean and unit variance.(2) Linear detrending and band-pass filtering (0.01–0.08 Hz). (3) Regression of 12 head-motion parameters and whole-brain signal. (4) Gaussian smoothing (sigma = 2.55 mm) on the 32 k fs_LR surface.

### Individualized homologous functional regions parcellation

Following preprocessing of fMRI data, individualized functional networks were parcellated, homologous networks were identified, and ROI-ROI functional connectivity was calculated using the Homologous Functional Regions Across Individuals toolbox (HFR_ai, http://nmr.mgh.harvard.edu/bid/DownLoad.html). Initially, the individual parcellation was conducted based on the methodology proposed by Wang et al. (2015). The confidence value was set to 3, with 10 terminating iterations, and homologous networks of the left and right hemispheres were combined. Subsequently, homologous functional regions across individuals were determined based on the individual parcellation results obtained from the previous step. The parcellation result after 10 consecutive iterations was selected, with a match rate set to 1, indicating that all subjects had matched that ROI or the kept ROI was homologous in function across all individuals. Finally, ROI-ROI functional connectivity was calculated using the homologous regions defined in the previous step.

### Individualized cortical functional networks spatial consistency evaluation

Intra-subject reliability and inter-subject variability were measured using the Dice coefficient. The Dice coefficient measures the similarity between two sets of data, such as binary masks or segmentations of an image. The Dice coefficient is defined as the ratio of the intersection or overlap of two sets of data to their union:

Dice coefficient = 2 |A ∩ B|/ (|A| + |B|). In this study, each individualized network was transformed into a binary mask. |A| + |B| indicates the sum of the number of total voxels within network A and B. And |A ∩ B|indicates the number of the common voxels between network A and B.

We used dice coefficient to describe the spatial consistency of cortical functional networks obtained by parcellation of BOLD images from different MRI devices, i.e., 3.0 T and 5.0 T field strengths. Thus there were two different dice coefficients for each network. One was the dice coefficient of 3.0 T images and 5.0 T images for the same subject, which we named the “dice coefficient intra-individual,” and the other was for the different subjects (namely dice coefficient for one subject’s 3.0 T images and every other subject’s 5.0 T images), which we named the “dice coefficient inter-individual.” We then evaluated the difference between these two dice coefficients. A high intra-individual dice coefficient would indicate high spatial consistency of a certain network at different magnetic fields for the same subject, and reflects the stability of the individualized brain functional parcellation method. The inter-individual variability of the cortical networks would lead to a lower dice coefficients for different subjects and further reflects the functional complexity of corresponding networks.

### Between-networks functional connectivity consistency analysis of individualized cortical networks

The total number of sub-regions obtained by HFR-ai parcellation (the second step of HFR-ai above) was calculated and analyzed for 3.0 T and 5.0 T images. We clustered these sub-regions according to the 18-networks (except for lateral ventricle system) they belonged to. These 18-networks were divided by the two hemispheres into 36 networks. Then the BOLD signals of the homologous functional regions clustered to the same network were averaged. The FC between these 36 networks was calculated using the averaged BOLD signals. Thus a functional connectivity matrix of size 36 × 36 was obtained for each subject for 3.0 T and 5.0 T images, respectively. We used the Euclidian distance (Frobenius Norm) to measure the consistency between two FC matrices. The Euclidian distance of two matrices was defined as the square root of the sum of the absolute squares of the difference between their corresponding elements:


$$dist\left(A,B\right)=\sqrt{\overset{n}{\underset{i=1}{\sum }}\overset{n}{\underset{j=1}{\sum }}{\left(a_{ij}-b_{ij}\right)}^{2}}$$

The intra-individual Euclidian distance between the 3.0 T network FC matrix and the 5.0 T network FC matrix was calculated for each subject. The inter-individual Euclidian distance between the 3.0 T network FC matrix of each subject and the 5.0 T network FC matrices of other subjects was also calculated.

### Graph theory analysis of functional connectivity matrices

For the between-network FC matrices (FC between the 36 networks) and the between sub-regions matrices (FC between the 75 sub-regions for 3.0 T and the 84 sub-regions for 5.0 T) obtained from HFR-ai parcellation, global network metrics were calculated using the Graph Theoretical Network Analysis (GRETNA).^1^ These global network metrics included small-world parameters such as the clustering coefficient (Cp), the characteristic path length (Lp), normalized clustering coefficient (γ), normalized characteristic path length (λ) and small-worldness (σ), as well as other global network metrics like global network efficiency, assortativity, synchronization, and hierarchy. Subsequently, the correlation of the global network metrics between 3.0 T and 5.0 T images for these two matrices was calculated to explore the consistency and stability of functional connectivity of individualized cortical functional networks across different magnetic field strengths.

### Statistical analysis

Statistical analyses were conducted using SPSS 23.0 software (SPSS, Inc., Chicago, IL, USA). A significance level of *p* < 0.05 was considered statistically significant. A paired sample t-test was used for the statistics of image SNR, tSNR and CNR between groups. A two-way Analysis of Variance (ANOVA) was used to calculate the main and interaction effects for individual and cortical network factors of the difference of dice coefficient. Group statistics of dice coefficients were compared using a two-sample independent t-test. The area under curve (AUCs) of all the network metrics of 3.0 T and 5.0 T images was statistically analyzed using Pearson correlation analysis.

## Results

### SNR, tSNR and CNR of 3.0 T and 5.0 T resting-state fMRI data

The original three cross-sectional views of raw fMRI images (axial, coronal and sagittal) of 3.0 T and 5.0 T of one representative subject were demonstrated (Supplementary Figure S2). The quality of 5.0 T images was visibly better than that of 3.0 T images. Significant statistical differences were observed in SNR (370.8 ± 61.8 vs. 144.8 ± 29.7, *p* < 0.0001) and CNR (0.88 ± 0.20 vs. 0.65 ± 0.10, *p* < 0.0001) between two groups (Supplementary Table S1).

The tSNR map calculated on a voxel-wise manner of 3.0 T and 5.0 T have been demonstrated (Supplementary Figure S3). The tSNR measured in a gray matter ROI of 5.0 T images was superior to 3.0 T (27.95 ± 2.66 vs. 25.78 ± 2.72, *p* < 0.0001). In addition, the water phantom tSNR of 5.0 T was also significantly better than that of 3.0 T (320.13 vs. 210.24) (Supplementary Table S1).

### Spatial consistency of individualized cortical functional parcellation between 3.0 T and 5.0 T images

**Figure 1:**
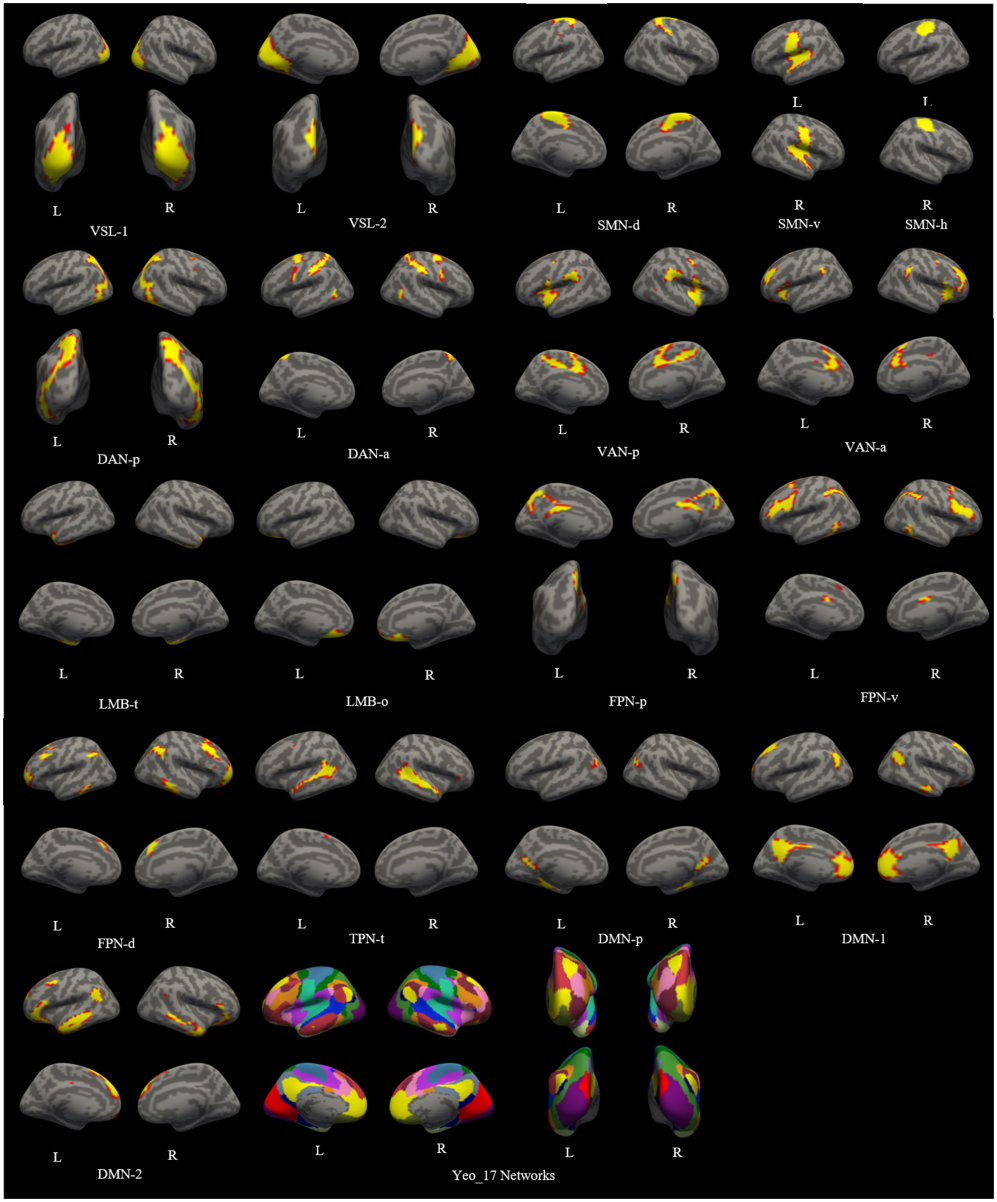
The functional cortical atlas consisting of 18 networks individually parcellated on both images of 3.0 T and 5.0 T. VSL, Visual Network; SMN-d, Dorsal Somatomotor Network; SMN-v, Ventral Somatomotor Network; SMN-h, Somatomotor Network for hand; DAN-p, Posterior Dorsal Attention Network; DAN-a, Anterior Dorsal Attention Network; VAN-p, Posterior Ventral Attention Network; VAN-a, Anterior Ventral Attention Network; LMB-t, Temporal Limbic Network; LMB-o, Orbitofrontal Limbic Network; FPN-p, Posterior Frontoparietal Network; FPN-v, Ventrolateral Frontoparietal Network; FPN-d, Dorsolateral Frontoparietal Network; TPN-t, Temporal Parietal Network; DMN, Default Network; DMN-p, Posterior Default Network.

A functional cortical atlas comprising 18 networks (excluding the lateral ventricle system) for each unilateral cerebral hemisphere was individually parcellated on both 3.0 T and 5.0 T images. The details of this template atlas was provided in the original paper (Yeo et al., 2011). The lateral ventricle, not belonging to the cortical network, was excluded from our study. The labels of these 18 networks and the atlas projected onto the individual cortical surface are depicted in Figure 1 (Wang et al., 2020).

**Figure 2:**
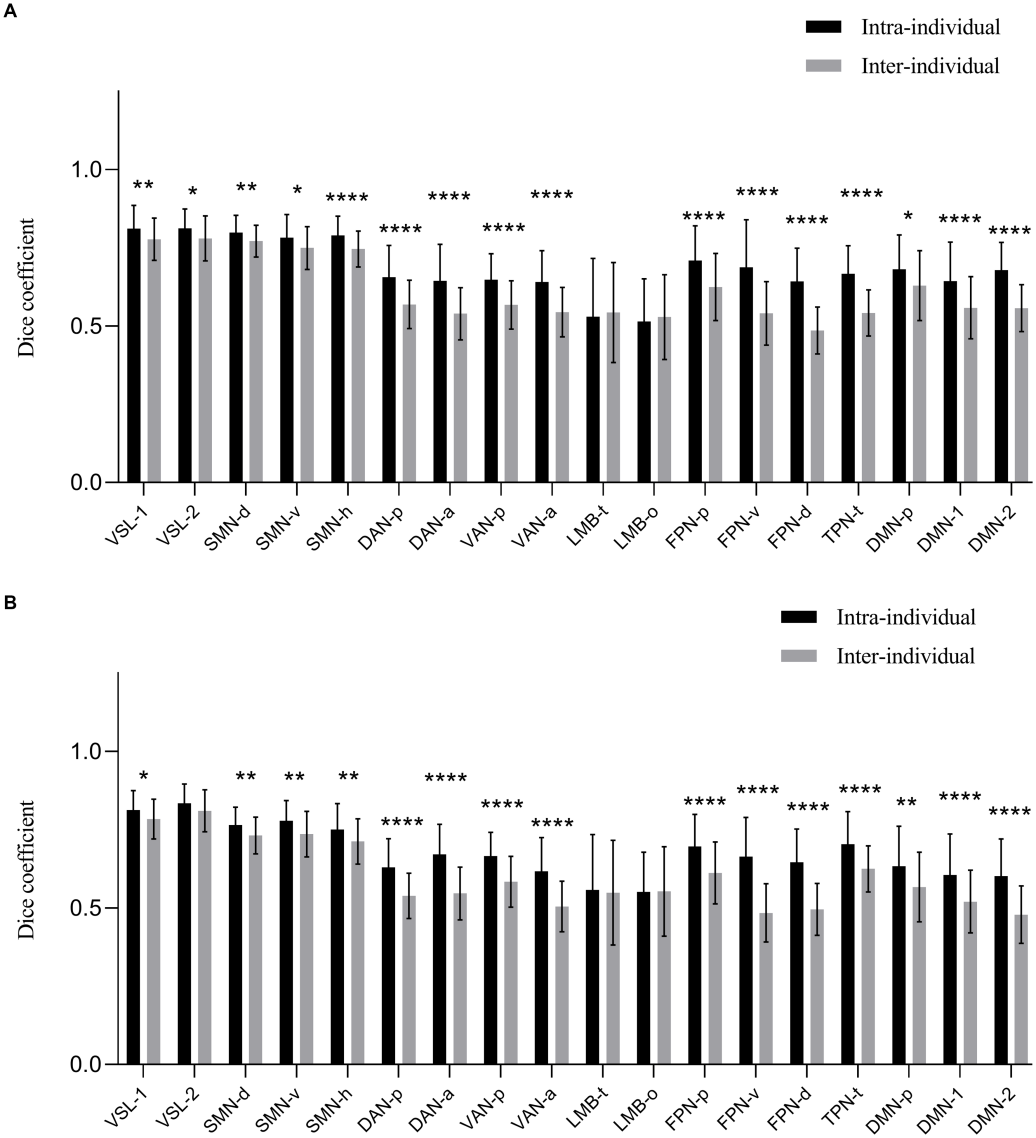
The dice coefficient of obtained 18 cortical networks of 3.0 T and 5.0 T images for intra and inter-individual. **(A)** The left hemisphere. **(B)** The right hemisphere. The dice coefficient of each network at 3.0 T and 5.0 T was generally high value (range: 0.5143–0.8337) of bilateral hemispheres. Almost all cortical functional networks had the higher dice coefficient intra-individual than that of inter-individual with significant statistic difference (*p* < 0.05), except for bilateral networks of LMB-t and LMB-o. VSL, Visual Network; SMN-d, Dorsal Somatomotor Network; SMN-v, Ventral Somatomotor Network; SMN-h, Somatomotor Network for hand; DAN-p, Posterior Dorsal Attention Network; DAN-a, Anterior Dorsal Attention Network; VAN-p, Posterior Ventral Attention Network; VAN-a, Anterior Ventral Attention Network; LMB-t, Temporal Limbic Network; LMB-o, Orbitofrontal Limbic Network; FPN-p, Posterior Frontoparietal Network; FPN-v, Ventrolateral Frontoparietal Network; FPN-d, Dorsolateral Frontoparietal Network; TPN-t, Temporal Parietal Network; DMN, Default Network; DMN-p, Posterior Default Network. *p < 0.05. **p < 0.01. ***p < 0.001. ****p < 0.0001.

The calculated dice coefficients of each networks are presented in Figure 2 and Table 1. The bilateral hemispheres were calculated separately. The intra-individual dice coefficient of each cortical functional network between 3.0 T and 5.0 T was high (range: 0.5143–0.8337) for both hemispheres. The results of ANOVA analysis revealed that the main and interaction effects of individual and cortical network factors on the dice coefficient were statistically significant (*F* > 8.3, *p* < 0.0001, see Supplementary Figure S4 and Supplementary Table S2). This suggests that individual and cortical network were main factors affecting the difference in dice coefficients. The post-hoc analysis at each cortical network showed that almost all cortical functional networks exhibited a higher dice coefficient intra-individually than inter-individually, with significant statistical differences (*p* < 0.05), except for bilateral temporal limbic network (LMB-t) and orbitofrontal limbic network (LMB-o).

**Table 1:** The Mean and SD of dice coefficient intra and inter-individual for 18 networks in bilateral hemispheres.

Network	Dice coefficient intra-individual			Dice coefficient inter-individual			
	Mean	SD	*N*	Mean	SD	*N*	*p*-value
VSL-1	0.8116^#^/0.8125^*^	0.0735^#^/0.0624^*^	30	0.7771^#^/0.7835^*^	0.0672^#^/0.0636^*^	870	0.0061^#^/0.0142^*^
VSL-2	0.8122/0.8337	0.0612/0.0621	30	0.7796/0.8102	0.0717/0.0666	870	0.0142/0.0579
SMN-d	0.7989/0.7642	0.0550/0.0574	30	0.7710/0.7316	0.0507/0.0586	870	0.0033/0.0028
SMN-v	0.7816/0.7783	0.0743/0.0646	30	0.7493/0.7358	0.0684/0.0727	870	0.0114/0.0016
SMN-h	0.7894/0.7499	0.0616/0.0835	30	0.7460/0.7122	0.0573/0.0722	870	<0.0001/0.0053
DAN-p	0.6562/0.6291	0.1013/0.0919	30	0.5689/0.5386	0.0768/0.0724	870	<0.0001/<0.0001
DAN-a	0.6445/0.6712	0.1162/0.0960	30	0.5393/0.5464	0.0833/0.0843	870	<0.0001/<0.0001
VAN-p	0.6484/0.6656	0.0822/0.0758	30	0.5674/0.5835	0.0770/0.0815	870	<0.0001/<0.0001
VAN-a	0.6412/0.6167	0.0991/0.1078	30	0.5440/0.5044	0.0789/0.0805	870	<0.0001/<0.0001
LMB-t	0.5298/0.5571	0.1858/0.1776	30	0.5430/0.5486	0.1597/0.1670	870	0.6584/0.7857
LMB-o	0.5143/0.5505	0.1359/0.1275	30	0.5285/0.5527	0.1355/0.1433	870	0.5741/0.9345
FPN-p	0.7092/0.6967	0.1110/0.1023	30	0.6243/0.6118	0.1073/0.0988	870	<0.0001/<0.0001
FPN-v	0.6874/0.6638	0.1518/0.1257	30	0.5401/0.4842	0.1014/0.0931	870	<0.0001/<0.0001
FPN-d	0.6426/0.6465	0.1062/0.1056	30	0.4854/0.4951	0.0750/0.0828	870	<0.0001/<0.0001
TPN-t	0.6661/0.7032	0.0900/0.1044	30	0.5413/0.6246	0.0738/0.0738	870	<0.0001/<0.0001
DMN-p	0.6811/0.6330	0.1094/0.1277	30	0.6289/0.5668	0.1113/0.1110	870	0.0117/0.0014
DMN-1	0.6439/0.6054	0.1241/0.1307	30	0.5581/0.5204	0.0993/0.1003	870	<0.0001/<0.0001
DMN-2	0.6787/0.6025	0.0886/0.1175	30	0.5567/0.4785	0.0750/0.0916	870	<0.0001/<0.0001

SD, Standard Deviation; N, Number; VSL, Visual Network; SMN-d, Dorsal Somatomotor Network; SMN-v, Ventral Somatomotor Network; SMN-h, Somatomotor Network for hand; DAN-p, Posterior Dorsal Attention Network; DAN-a, Anterior Dorsal Attention Network; VAN-p, Posterior Ventral Attention Network; VAN-a, Anterior Ventral Attention Network; LMB-t, Temporal Limbic Network; LMB-o, Orbitofrontal Limbic Network; FPN-p, Posterior Frontoparietal Network; FPN-v, Ventrolateral Frontoparietal Network; FPN-d, Dorsolateral Frontoparietal Network; TPN-t, Temporal Parietal Network; DMN, Default Network; DMN-p, Posterior Default Network. ^#^Left hemisphere. ^*^Right hemisphere.

Based on the intra-individual dice coefficient, along with the statistical differences between dice coefficient intra and inter-individuals, we classified all parcellated cortical functional networks into three categories. First, networks with a relatively high intra-individual dice coefficients and slight statistical difference between intra and inter-individual dice coefficients. These included bilateral visual network-1 (VSL-1), Visual Network-2 (VSL-2), dorsal somatomotor network (SMN-d), ventral somatomotor network (SMN-v) and somatomotor network for hand (SMN-h) (intra-individual dice coefficient > 0.75). Second, networks with moderate dice coefficient intra-individual and significant statistical difference between intra and inter-individual dice coefficients were identified. These encompassed networks such as bilateral posterior dorsal attention network (DAN-p), anterior dorsal attention network (DAN-a), posterior ventral attention network (VAN-p), anterior ventral attention network (VAN-a), posterior frontoparietal network (FPN-p), ventrolateral frontoparietal network (FPN-v), dorsolateral frontoparietal Network (FPN-d), temporal parietal network (TPN-t), posterior default network (DMN-p), default network-1 (DMN-1) and default network-2 (DMN-2) (intra-individual dice coefficients 0.60–0.75). Third, the networks with relatively lower dice coefficient both intra-and inter-individual. The remaining networks of bilateral LMB-t and LMB-o fell into this category (intra-individual dice coefficient 0.50–0.60).

### The number of parcellated individualized cortical functional sub-regions for 3.0 T and 5.0 T images

**Figure 3:**
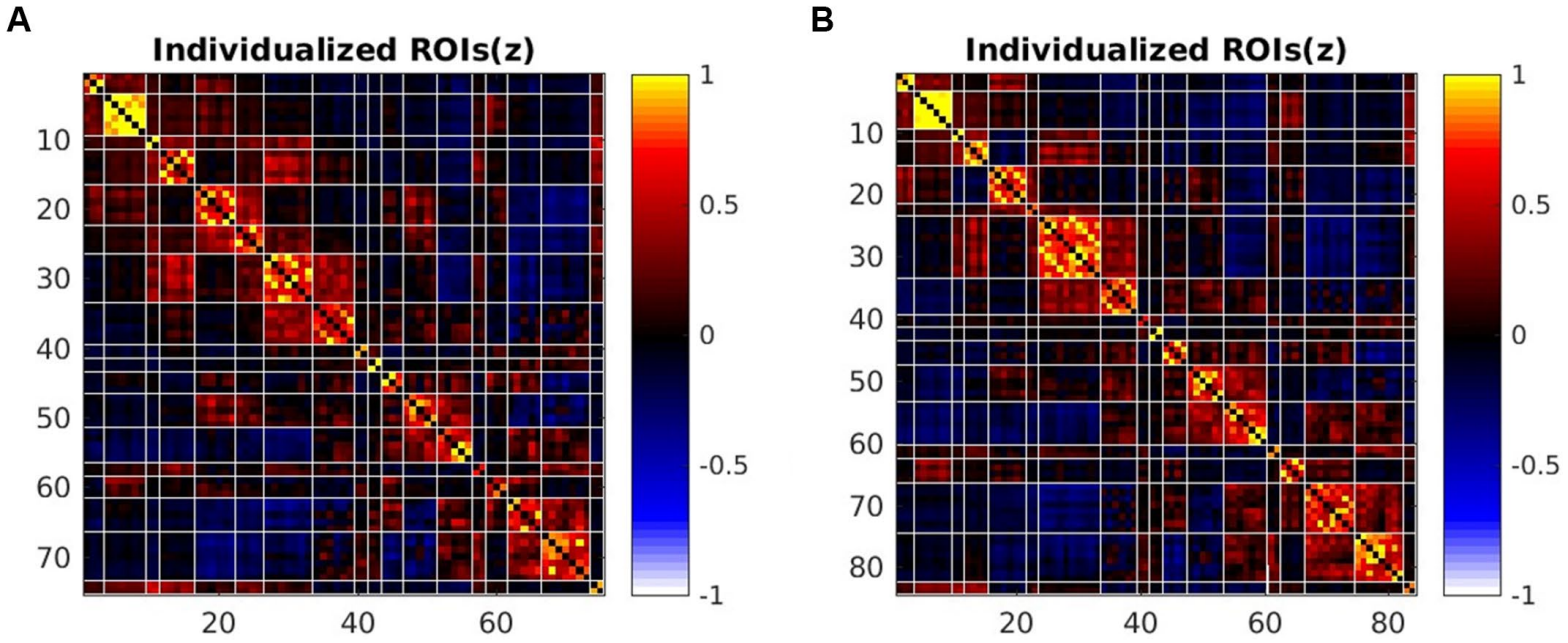
The average FC matrix of sub-regions at 3.0 T and 5.0 T by HFR software. **(A)** The 75*75 sub-regions FC matrix of 3.0 T images. **(B)** The 84*84 sub-regions FC matrix of 5.0 T images.

**Figure 4:**
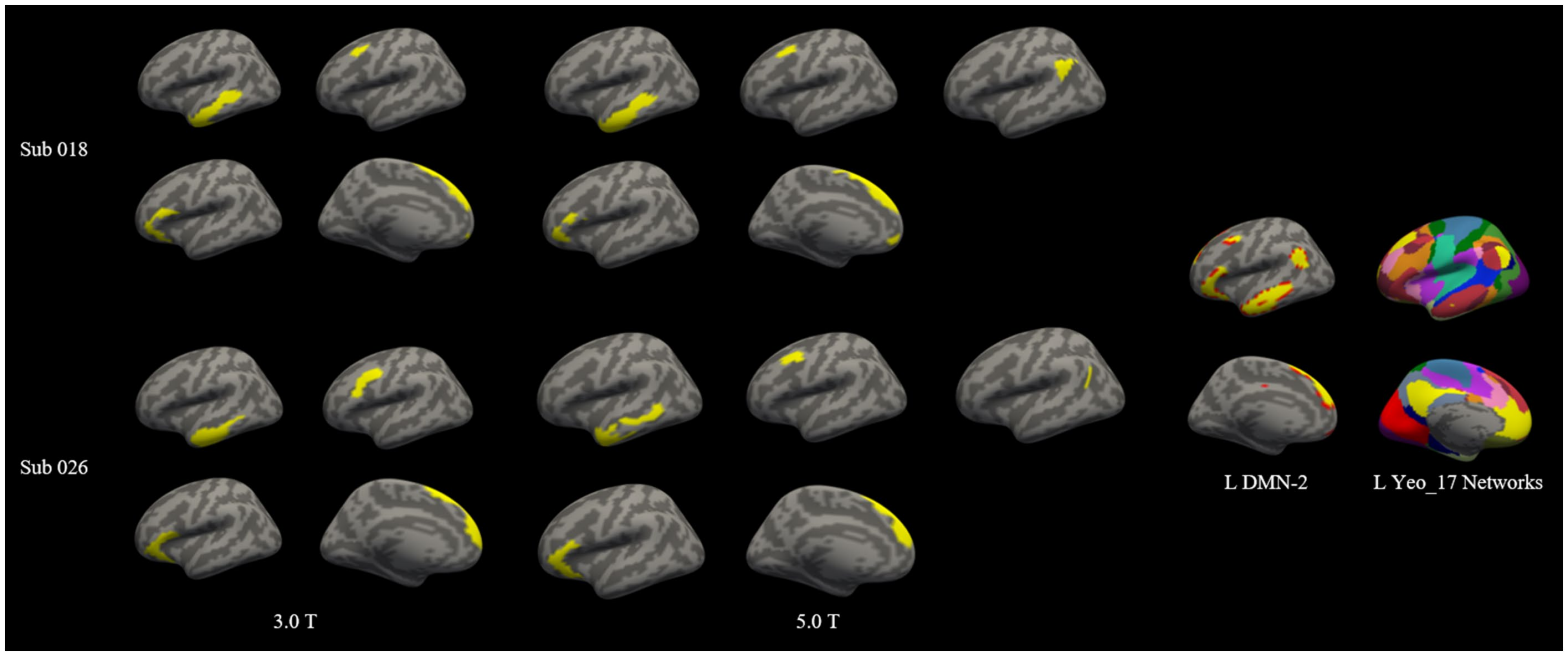
Two representative subjects with high and moderate dice coefficients for the left DMN-2 network at 3.0 T and 5.0 T. Subject 018 was with high dice coefficient (0.8084) and subject 026 was with moderate dice coefficient (0.6119) intra-individually. The individualized left DMN-2 networks were projected onto the individual cortical surface. The spatial distribution of left DMN-2 network was highly similar between 3.0 T and 5.0 T for subject 018, however the spatial distribution of this network was slightly different on 3.0 T and 5.0 T for subject 026. Note that the angular gyrus was correctly identified on 5.0 T but was missing on 3.0 T, hence the number of the parcellated sub-regions of DMN-2 was 5 at 5.0 T, and 4 at 3.0 T.

The total number of individualized homologous functional sub-regions was 75 for 3.0 T images and 84 for 5.0 T images (Figure 3 and Table 2). Each of the sub-regions was displayed for a representative individual (Supplementary Figure S5). These functional regions constituted the 36 larger brain networks of bilateral cerebral hemispheres. For each of the 36 parcellated networks, the number of functional sub-regions parcellated was shown in Table 2. Figure 4 demonstrated two representative individuals, and it clear that the angular gyrus was correctly identified on 5.0 T but was missing on 3.0 T for both subjects, hence the number of the parcellated sub-regions of DMN-2 was 5 at 5.0 T, and 4 at 3.0 T (Figure 4). Likely, in some other networks (e.g., right VSL-1, bilateral VAN-p, left FPN-p, left FPN-v, bilateral FPN-d, right DMN-p, bilateral DMN-1, and left DMN-2), the number of sub-regions parcellated from 5.0 T images exceeded that from 3.0 T images.

**Table 2:** The number of sub-regions parcellated by HFR of 3.0 T and 5.0 T images.

Network	3.0 T		5.0 T	
	Left hemisphere	Right hemisphere	Left hemisphere	Right hemisphere
VSL-1	2	1	1	2
VSL-2	3	3	3	3
SMN-d	1	1	1	1
SMN-v	3	2	2	2
SMN-h	1	1	1	1
DAN-p	3	3	3	3
DAN-a	2	2	1	1
VAN-p	3	4	5	5
VAN-a	3	3	3	3
LMB-t	1	1	1	1
LMB-o	1	1	1	1
FPN-p	1	2	2	2
FPN-v	3	2	4	2
FPN-d	2	3	3	4
TPN-t	1	1	1	1
DMN-p	2	1	2	2
DMN-1	3	2	4	4
DMN-2	4	3	5	3
Total	39	36	43	41

VSL, Visual Network; SMN-d, Dorsal Somatomotor Network; SMN-v, Ventral Somatomotor Network; SMN-h, Somatomotor Network for hand; DAN-p, Posterior Dorsal Attention Network; DAN-a, Anterior Dorsal Attention Network; VAN-p, Posterior Ventral Attention Network; VAN-a, Anterior Ventral Attention Network; LMB-t, Temporal Limbic Network; LMB-o, Orbitofrontal Limbic Network; FPN-p, Posterior Frontoparietal Network; FPN-v, Ventrolateral Frontoparietal Network; FPN-d, Dorsolateral Frontoparietal Network; TPN-t, Temporal Parietal Network; DMN, Default Network; DMN-p, Posterior Default Network.

### Consistency of between-network functional connectivity of individualized cortical networks between 3.0 T and 5.0 T images

**Figure 5:**
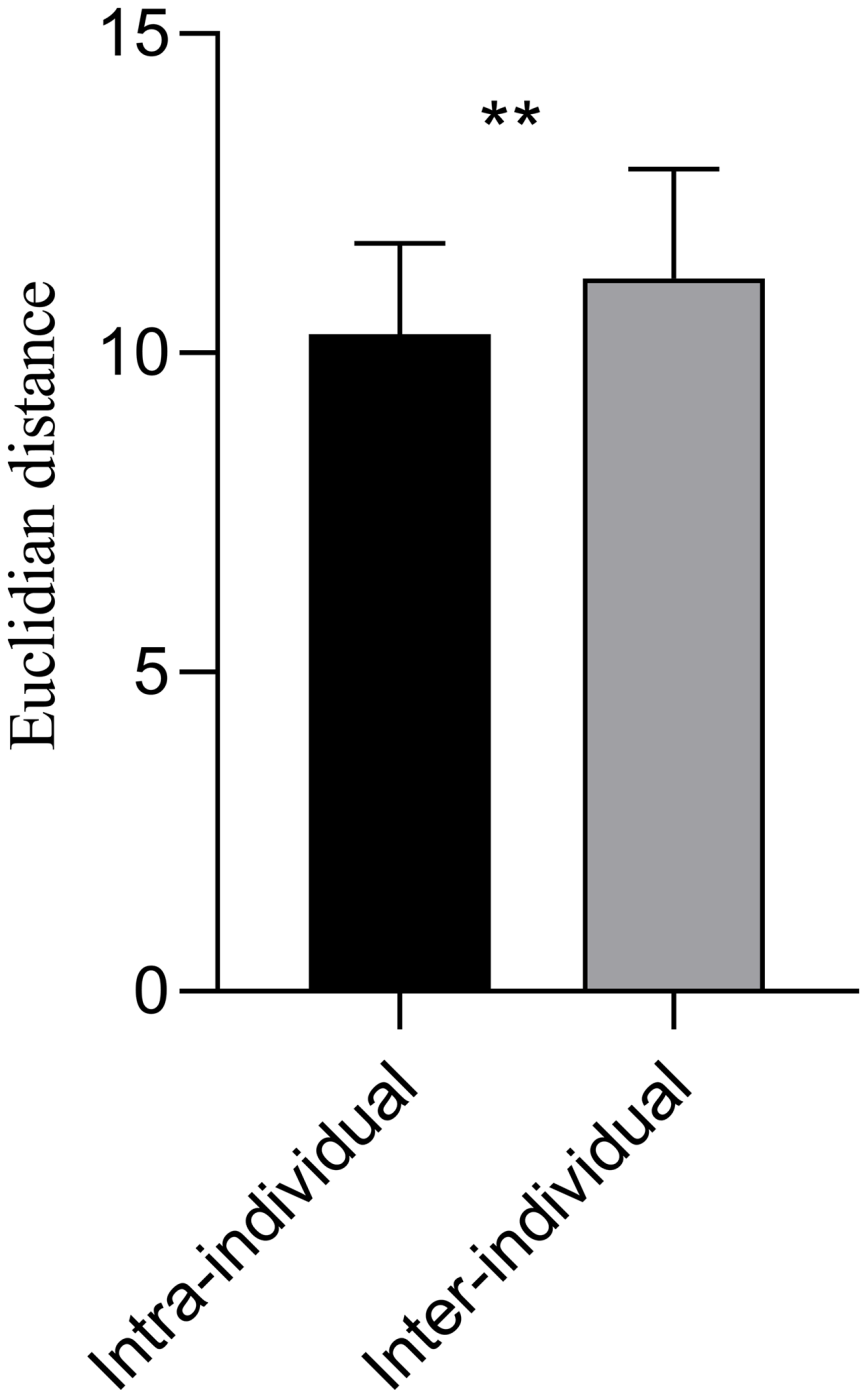
The Euclidian distance of the 36 × 36 averaged FC matrix at 3.0 T and 5.0 T for intra and inter-individual. The mean Euclidian distance for inter-individual was higher than that of intra-individual (11.1647 ± 1.7128 vs. 10.2922 ± 1.4312). Significant statistical difference intra and inter-individuals on the 3.0 T and 5.0 T images was observed (*p* = 0.006). ^**^p < 0.01.

Significant statistical differences were observed between intra and inter-individuals for the Euclidean distance of the between-network FC matrices (*p* = 0.006) (Figure 5). The mean Euclidian distance for inter-individuals was higher than that of intra-individuals (11.1647 ± 1.7128 vs. 10.2922 ± 1.4312).

### Graph theory metrics consistency

**Figure 6:**
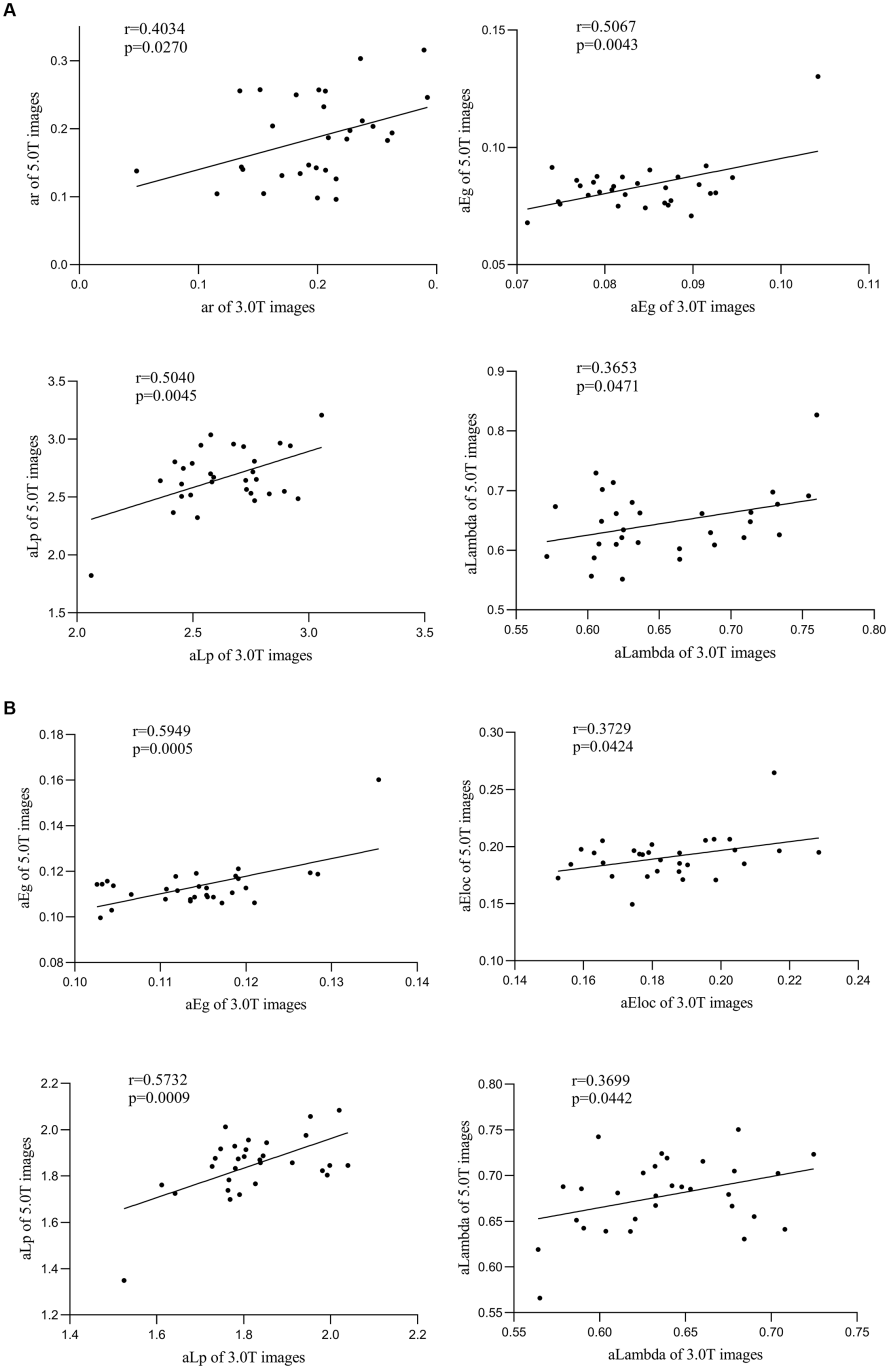
Correlation analysis of functional connectivity matrix of individualized cortical networks between 3.0 T and 5.0 T images. **(A)** For the 36 × 36 averaged functional connectivity matrix between the 3.0 T and 5.0 T images, a positive correlation in metrics of assortativity (ar, *r* = 0.403, *p* = 0.027), network efficiency (aEg, *r* = 0.507, *p* = 0.004), characteristic path length (aLambda, *r* = 0.365, *p* = 0.047) and normalized characteristic path length (aLp, *r* = 0.504, *p* = 0.005) has been observed. **(B)** For the primitive sub-regions matrix, significant positive correlation between 3.0 T and 5.0 T images of network n (aEg, *r* = 0.595, *p* = 0.001; aEloc, *r* = 0.373, *p* = 0.042), characteristic path length (aLambda, *r* = 0.370, *p* = 0.044) and normalized characteristic path length (aLp, *r* = 0.573, *p* = 0.001) was also observed.

For correlation analysis of the global network metrics of the between-network FC matrices (size of 36 × 36) between the 3.0 T and 5.0 T images, a positive correlation was observed in metrics such as assortativity (ar, *r* = 0.403, *p* = 0.027), network efficiency (aEg, *r* = 0.507, *p* = 0.004), characteristic path length (aLambda, *r* = 0.365, *p* = 0.047), and normalized characteristic path length (aLp, *r* = 0.504, *p* = 0.005) (Figure 6A).

Similarly, a significant positive correlation of certain global network metrics was observed in the between-sub-regions matrices (matrix size of 75 × 75 for 3.0 T and matrix size of 84 × 84 for 5.0 T) images obtained from HFR-ai parcellation on (Figure 6B), namely network efficiency (aEg, *r* = 0.595, *p* = 0.001; aEloc, *r* = 0.373, *p* = 0.042), characteristic path length (aLambda, *r* = 0.370, *p* = 0.044) and normalized characteristic path length (aLp, *r* = 0.573, *p* = 0.001).

## Discussion

Parcellating functional networks across the cerebral cortex in individuals based on functional connectivity is particularly important for personalized diagnosis and treatment (Fan et al., 2020; Gordon et al., 2022). This study represents the first attempt to evaluate the consistency and stability of individualized cortical functional networks parcellation at 3.0 T and 5.0 T MRI. We delineated 18 cortical functional networks based on a previously proposed cortical parcellation algorithm and derived several key findings (Wang et al., 2015). Firstly, the spatial consistency (dice coefficient) of the parcellated 18 networks between 3.0 T and 5.0 T within the same individuals was generally high, surpassing that of inter-individuals. Secondly, the total number of sub-regions obtained by individualized parcellation of cerebral cortex at 5.0 T exceeded that of 3.0 T, indicating a higher sensitivity and enhanced cross-regional contrast achievable at 5.0 T. Thirdly, we observed high consistency of individualized FC between 3.0 T and 5.0 T for the same subject, indicated by a significantly lower Euclidian distance of the between-network FC matrix between 3.0 T and 5.0 T intra-individual than inter-individual. Fourthly, for graph theory analysis of the individualized FC, we identified a significant correlation in graph theory metrics (such as assortativity, network efficiency, and small-worldness) between 3.0 T and 5.0 T. These results helped verify the consistency and stability of individualized cortical functional networks parcellation at 3.0 T and 5.0 T, and demonstrated the variance of cortical networks across individuals (Laumann et al., 2015).

All the parcellated cortical functional networks could be classified into three categories based on the intra-and inter-individual dice coefficients between 3.0 T and 5.0 T. First, the category with a relatively high dice coefficient intra-individual and slight statistic difference between dice coefficient intra and inter-individual. The spatial consistency of these cortical networks within individuals was very high across different magnetic fields, and the spatial variance between individuals was relatively small. The parcellated cortical functional networks of bilateral VSL-1, VSL-2, SMN-d, SMN-v and SMN-h were classified into this category. Previous FC study also revealed that SMN and VSL systems had low inter-subject variability (Mueller et al., 2013), consistent with our findings. Both VSL and SMN are primary sensory and motor networks. The low variabilities of VSL and SMN was related to their dedicated and simpler functions, and their less complicated evolutionary cortical expansion and cortical folding (Hill et al., 2010). Interestingly, we have recognized a new network not labeled independently in Yeo’s 17-network atlas. It was located in the anterior central gyrus (the part of the brain that responsible for hand movement) and was part of the somatomotor network, we named it SMN-h. Quite significant differences between intra and inter-individuals were demonstrated in SMN-h in left hemisphere, but not in SMN-d or SMN-v, nor in the right hemisphere. This provided two implications. First, compared to other SMN sub-networks, SMN-h has more complex functions and greater individual variability. Second, in terms of lateralization, SMN-h in the left hemisphere exhibited more complicated functional activities than that of right hemisphere. This probably can be explained by the fact that the healthy participants included in our study were all right-handed.

The second category of networks showed moderate intra-individual dice coefficients and significant statistical difference between intra-and inter-individual dice coefficients. These networks include the bilateral DAN-p, DAN-a, VAN-p, VAN-a, FPN-p, FPN-v, FPN-d, TPN-t, DMN-p, DMN-1 and DMN-2. The DAN is supposed to be organized bilaterally and comprises the intraparietal sulcus (IPS), the frontal eye fields (FEF) and superior parietal lobule of each hemisphere (Petersen and Posner, 2012; Vossel et al., 2014). The VAN is mainly located in Temporal–parietal junction (right-lateralized) and ventral prefrontal cortex (Vossel et al., 2014), generally considered part of the Salience network. Both DAN and VAN are higher-order cognitive networks, previous studies have shown great variability of these two networks by using different parcellation models (Vossel et al., 2014; Allan et al., 2020; Ramezanpour and Fallah, 2022). FPN is a key component of higher order function network and has extensive connectivity with many different brain networks, making it a functional hub of cognitive functions (Power et al., 2013). The study of precise mapping reflected the large individual variation in the precise anatomy of the FPN (Laumann et al., 2015). In addition, previous study also demonstrated FPN and attentional networks showed high inter-individual variability (Mueller et al., 2013), which was consistent with our results. The studies of DMN are the most extensive (Buckner, 2012). Related experiments analysis of intrinsic connectivity combined with graph-analytic and clustering techniques have demonstrated that DMN comprises two subsystems that interact with a common core, and the subsystems functionally dissociate when under different task or resting-state conditions (Andrews-Hanna et al., 2010). The results of our individualized analysis on different MR fields also confirmed the existence of three sub-networks of DMN. All the above networks are higher-order cognitive networks, thus it is not surprising that these networks showed significant inter-individual variability also less intra-individual consistency than the first category of visual and sensory-motor networks, indirectly reflecting their possible complex functions.

The sole cortical network classified into the third category with a relatively lower intra-and inter-individual dice coefficient was the limbic network (LMB-t and LMB-o). The limbic system can be divided into cortical and sub-cortical limbic regions. While the two sub-networks (LMB-o and LMB-t) we have parcellated seemed to mainly located in other extended nodes referred as the “limbic forebrain” (Morgane et al., 2005). The limbic system has been implicated in memory and mood regulations, and its status may change rapidly according to the subjects’ mood status. Our study suggested that these two networks were highly variable both intra-individual and across individuals, likely due to their involvement in short-term memory processing and mood-related functional activities. Besides, the limbic system areas are close to the bottom of the brain and are very susceptible to low SNR and signal drop, which might be one of the reasons that contributes to the low spatial consistency (dice coefficients) both intra and inter-individually of this network. However, by a visual check of the raw fMRI data (see Supplementary materials), we did not find any significant artefact or distortion of the medial and inferior temporal areas, despite a slightly weaker signal at the temporal pole. Thus, the inter-and intra-variability of the LMB networks is more likely due to the quick neurophysiological changes in these areas.

The total number of individualized homologous functional regions of 5.0 T images was slightly larger than that of 3.0 T images. In 11 of the 36 networks, more sub-regions were parcellated from 5.0 T images, predominantly in networks of FPN, DMN and VAN. This may be due to the superior image quality, higher SNR and CNR of 5.0 T than 3.0 T, and the spatially adjacent but functionally segregated sub-regions are more distinguishable at 5.0 T. We have compared the SNR, tSNR and CNR of the raw resting-state fMRI data between 3.0 T and 5.0 T groups. Results showed that the raw BOLD images at 5.0 T have higher SNR, tSNR and CNR than that at 3.0 T. This was expectable and consistent with previous studies (Triantafyllou et al., 2005; Schafer et al., 2008). The attenuation of intravascular signal from veins at higher field facilitated spatial specificity of detecting neural activity related signal (Gati et al., 1997). Studies on 7.0 T MR have shown that ultra-high field provides significant advantage over lower (3.0 T) field for functional connectivity measurement, based on higher BOLD CNR yielding higher temporal correlation between functionally connected brain areas (Duong et al., 2002; Hale et al., 2010). Another study used increased BOLD contrast-to-noise ratio at 7.0 T to measure the topographic representation of the digits in human somatosensory cortex at 1 mm isotropic resolution in individual subjects, this is almost impossible to achieve at or below 3.0 T field strength (Sanchez-Panchuelo et al., 2010). These results further confirmed that MRI with ultra-high field could obtain finer functional sub-network characteristics.

We further analyzed the consistency of the parcellated networks from a functional connectivity perspective. The mean Euclidian distance of FC matrices for inter-individual at 3.0 T and 5.0 T was significantly higher than for intra-individual. This indicated that functional connectivity between the 36 brain networks obtained by individualized parcellation was consistent within individuals across 3.0 T and 5.0 T, but variable across individuals. Multiple previous studies have also demonstrated the variability of the human cortical spontaneous activity across space, rs-fMRI platforms (3 T and 7 T), and individuals, with higher connectivity variability between participants and the lower connectivity variability within individual participants (Frost and Goebel, 2012; Xing et al., 2023). These findings elucidate individual variances in functional connectivity of cortical networks, and were again validated by our use of individualized parcellation method in high and ultra-high fields.

Correlation analysis of the graph theory global metrics for the between-network FC and sub-regions FC matrix obtained from HFR-ai parcellation showed a positive correlation between the 5.0 T and the 3.0 T matrices. The graph theory analysis results were consistent and correlated under different field strengths. A previous study have reported good or excellent test–retest reliability for many metrics including assortativity, characteristic path length, global efficiency and local efficiency at 3.0 T (Welton et al., 2015). Our results expanded these findings to different magnetic fields.

Several limitations in our study should be acknowledged. First, we set a total of 10 iterations in individualized parcellation based on previous reference (Wang et al., 2015). However, at 5.0 T, 10 iterations may not capture all individualized parcellation details. Thus probably resulting in fewer sub-regions parcellated for some networks at 5.0 T than 3.0 T. In the future, we intend to set more iterations at 5.0 T to evaluate the performance of individualized parcellation. Second, we set the same TE values of 25 ms and flip angle of 90° at 5.0 T MR with 3.0 T. These TE and flip angle values were very commonly used parameter settings, while it may not be the optimal for the 5.0 T field strength. For 5.0 T images, the optimal TE and Ernst angle is not clear and have not been reported. Yacoub et al. have reported TE = 25 ms was the optimal TE to be used in 7.0 T BOLD fMRI in the human visual cortex, while 35 ms was optimal TE at 4.0 T (Yacoub et al., 2001). Gonzalez-Castillo et al. have reported that for the gray matter compartment, the Ernst angle was 77° at 3.0 T BOLD fMRI. While using lower flip angles in 3.0 T BOLD fMRI experimentation may have benefits such as reduction of RF power, limitation of apparent T1-related inflow effects, reduction of through-plane motion artifacts and lower levels of physiological noise (Gonzalez-Castillo et al., 2011). More studies are needed to explore the optimal parameter for the BOLD images at 5.0 T MR. Third, we did not collect multiple resting-state scanning sessions at 3.0 T and 5.0 T. Previous study has confirmed the consistency of individualized parcellation at 3.0 T, indicating that the consistency and stability were reliable on the same field strength (Wang et al., 2015). Considering the purpose of our study was to explore the consistency and stability of individualized parcellation across different magnetic fields, only one session was collected for each field. Finally, we investigated the individual-specific functional connectivity by resting-state fMRI, while task-evoked conditions need further evaluation to draw similar conclusions.

In conclusion, we have confirmed that individualized cortical functional networks at different magnetic fields had consistent and stable parcellation results both spatially and functionally. The 5.0 T BOLD fMRI has higher SNR and CNR than 3.0 T and provides finer functional sub-network characteristics of individualized cortical functional networks parcellation. These results have laid the foundation for future application of individualized cortical functional networks parcellation at 5.0 T and other ultra-high field MR.

## Data Availability

The raw data supporting the conclusions of this article will be made available by the authors, without undue reservation.

## Footnotes

^1^https://www.nitrc.org/projects/gretna
