# Supplementary material for: Consistency and stability of individualized cortical functional networks parcellation at 3.0 T and 5.0 T MRI
Source: Front Neurosci. 2024 Aug 19;18:1425032. doi: 10.3389/fnins.2024.1425032 (PMC11366602; doi:10.3389/fnins.2024.1425032)
Supplement: Supplementary file 1 [file Data_Sheet_1.docx]

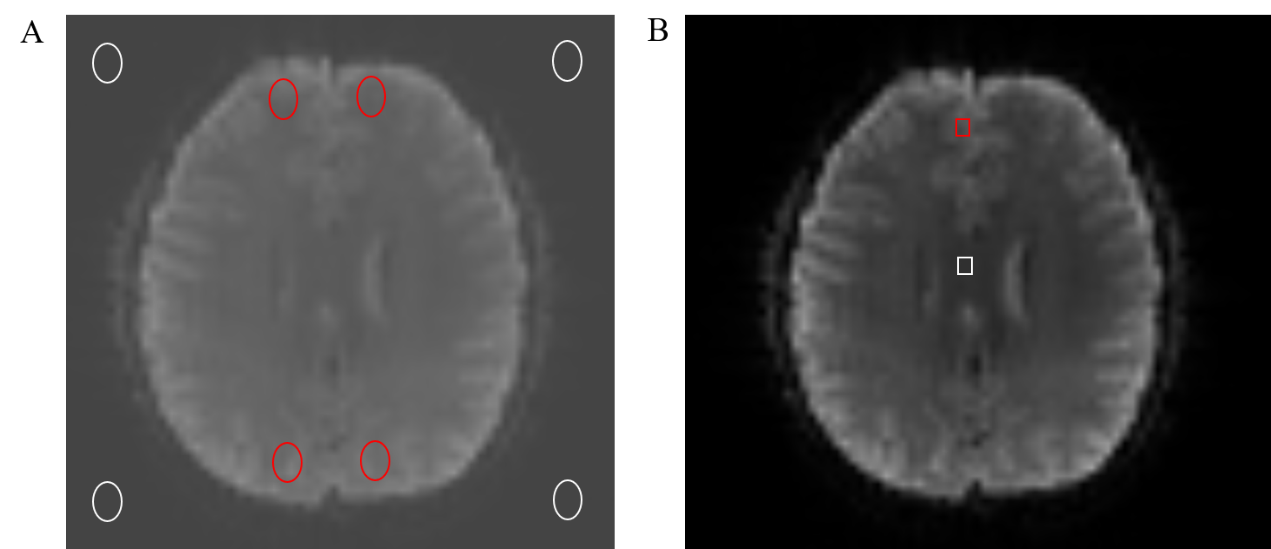


Supplementary Figure S1. The ROIs for SNR and CNR measurements. (A) demonstrated the ROIs of SNR were symmetrically drawn. The signal ROIs in frontal and parietal lobes at the apex level of the lateral ventricle (red ellipse) and the noise ROIs in the four corners of the image (white ellipse). Note that the color bar of the overall image was adjusted so as to show the background noise. (B) demonstrated the ROIs for CNR measurement. The ROI of gray matter was drawn on frontal lobe at the apex level of the lateral ventricle from axial conventional resting-state fMRI data (red rectangle), and ROI of white matter was drawn in the callosum area of the same level (white rectangle).


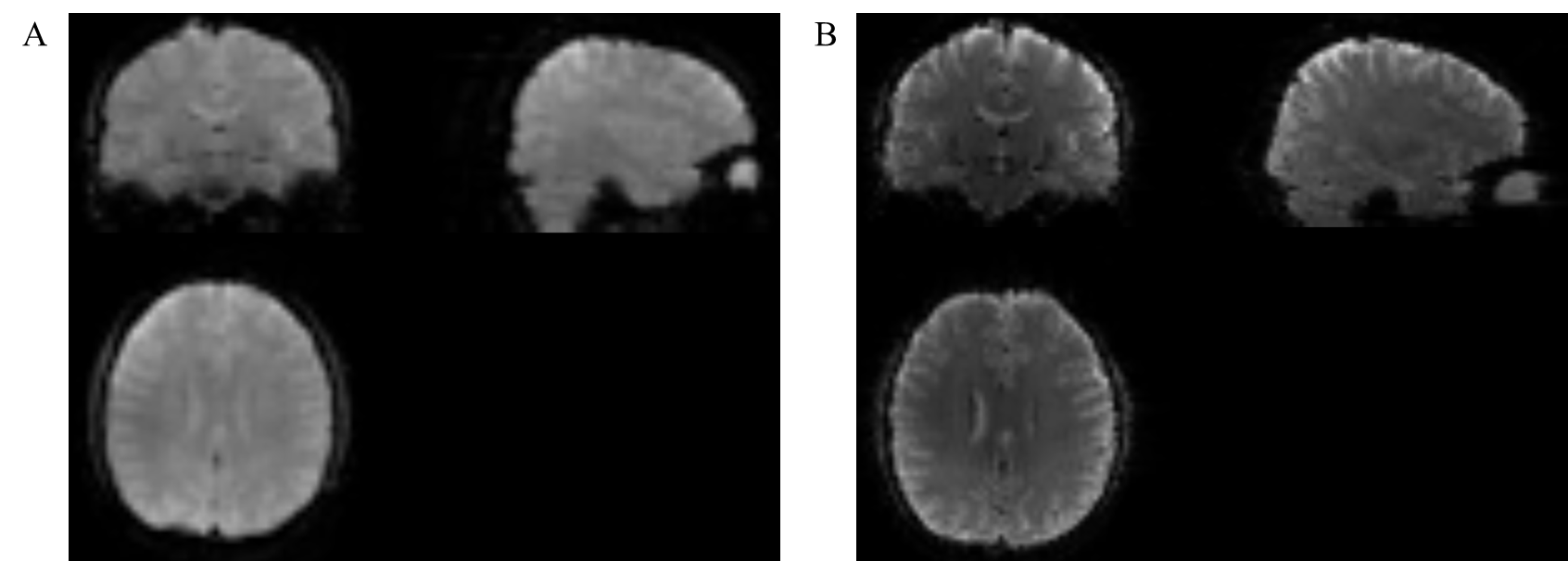


Supplementary Figure S2. The original three cross-sectional views of raw fMRI data (axial, coronal and sagittal) from 3.0 T and 5.0 T. (A) for 3.0 T and (B) for 5.0 T.


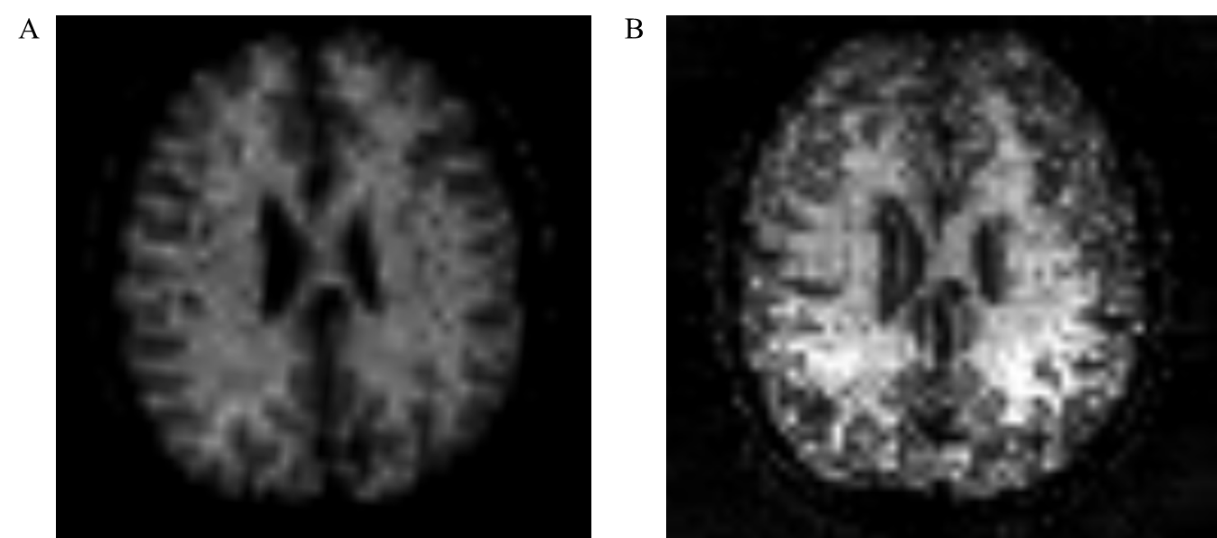


Supplementary Figure S3. The tSNR maps on a voxel-wise basis of one subject at 3.0 T and 5.0 T. (A) for 3.0 T and (B) for 5.0 T.


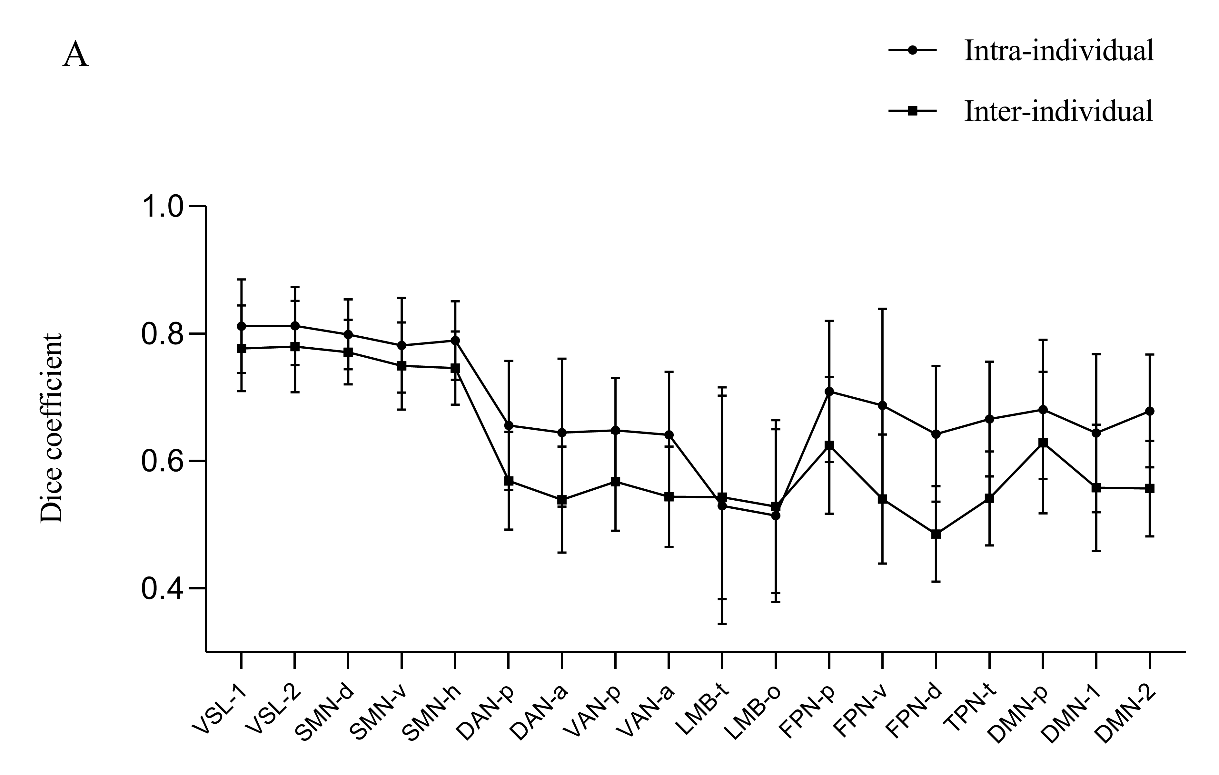


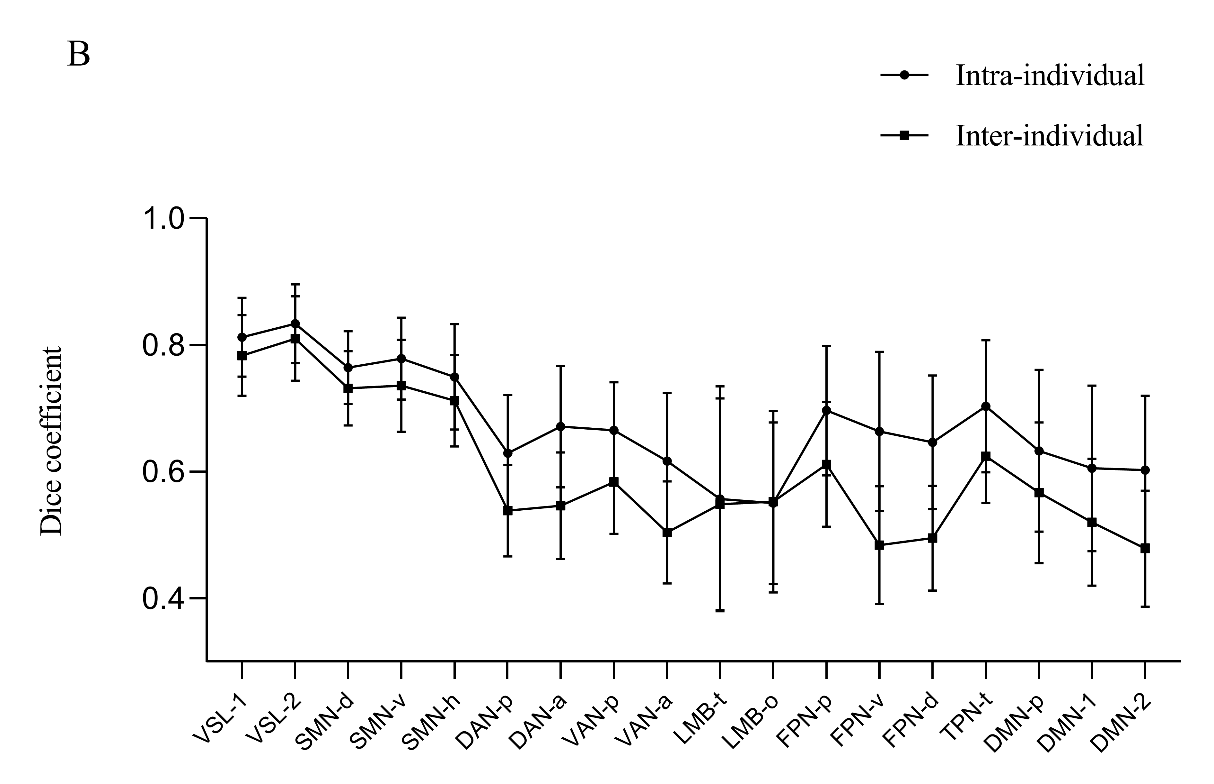


Supplementary Figure S4. The interaction effects of individual and cortical network factors on dice coefficient. (A) for left hemisphere and (B) for right hemisphere. Significant interactions of the individual effect and network effect were both seen in the left and right hemisphere.


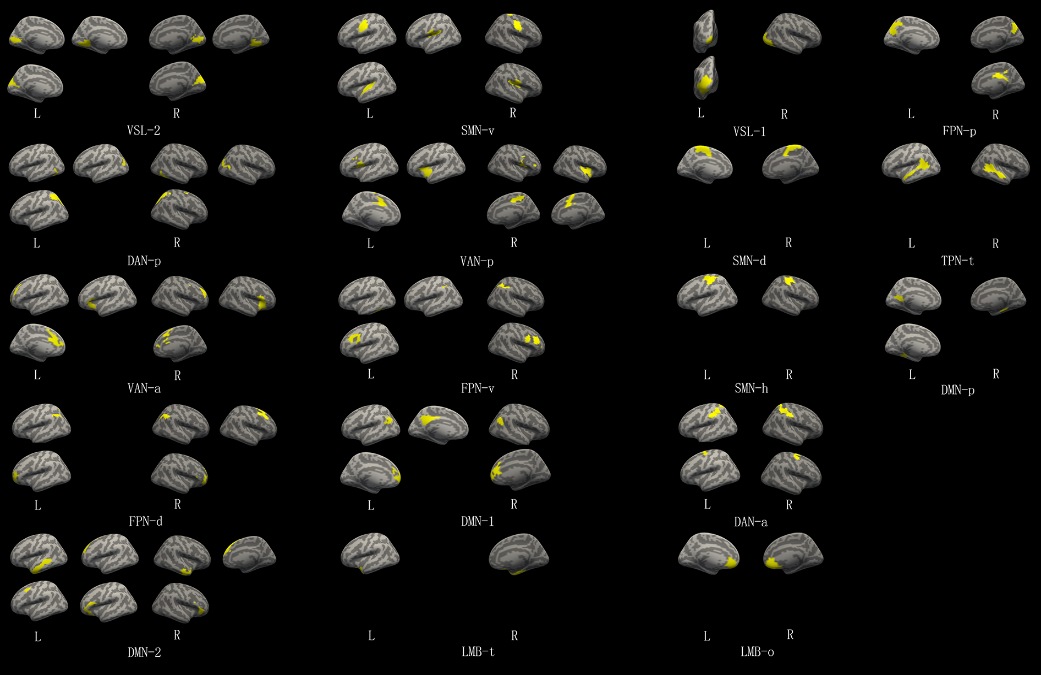

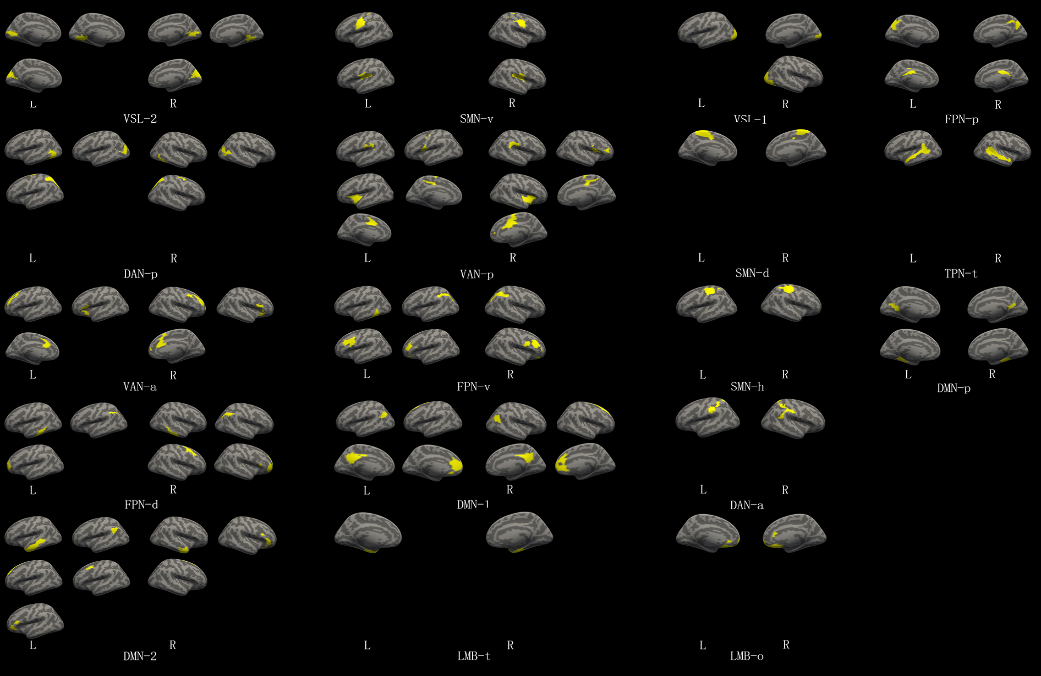


A B

Supplementary Figure S5. The atlas of 75 individualized homologous functional sub-regions for 3.0 T images and 84 for 5.0 T images of one representative subject. (A) for 3.0 T image (B) for 5.0 T image.

Supplementary Table S1. SNR, CNR and tSNR of raw resting-state fMRI data of 3.0 T and 5.0 T

|  | **3.0 T** | **5.0 T** | **5.0 T/3.0 T** | **P-value** |
| --- | --- | --- | --- | --- |
| SNR | 144.8±29.7 | 370.8±61.8 | 2.65±0.60 | <0.0001 |
| CNR | 0.65±0.10 | 0.88±0.20 | 1.37±0.37 | <0.0001 |
| tSNR | 25.78±2.72 | 27.95±2.66 | 1.09±0.08 | <0.0001 |
| tSNR_W_ | 210.24 | 320.13 | 1.52 | - |

SNR= Signal-to-Noise Ratio; CNR= Contrast-to-Noise Ratio; tSNR = temporal SNR; tSNR_W_ = temporal SNR of water film.

Supplementary Table S2. The two-way ANOVA analysis results of individual and cortical network factors on dice coefficient.

| **Factors** | **F-value** | **P-value** |
| --- | --- | --- |
| Network | 114.6^#^/112.0^*^ | <0.0001^#^/<0.0001^*^ |
| Individual | 317.4/332.6 | <0.0001/<0.0001 |
| Interaction | 8.708/8.303 | <0.0001/<0.0001 |

# Left hemisphere; * Right hemisphere.
